# THE PICTURE WITHOUT THE THOUSAND WORDS—PHYSICIAN AND JUDGE COMMUNICATION IN GUARDIANSHIP CASES

**DOI:** 10.1093/geroni/igad104.2260

**Published:** 2023-12-21

**Authors:** Ronan Factora, Matthew Pawlicki

**Affiliations:** Cleveland Clinic, Cleveland, Ohio, United States; University Hospitals, Chardon, Ohio, United States

## Abstract

In Ohio, physicians conducting capacity evaluations in guardianship cases are also asked to complete a Statement of Expert Evaluation (SEE) used by Probate Court to aid in decisions determining that person’s competence. In a prior survey, physicians voiced reluctance to conduct such evaluations because they were time consuming and judges stated that information provided by physicians in guardianship cases was inadequate or incomplete. Communication between the two groups of what information would be most useful in this process would help optimize the time required by physicians to complete their evaluation and improve the quality of information judges receive when making their decision. A survey was conducted of legal professionals (at an Ohio Probate Court Pre-conference session specifically focused on capacity evaluation and SEE completion) which sought to identify elements these legal professionals felt were most and least useful from SEEs provided by completing physicians. With 52 attendees (including judges (active and retired), magistrates, court personnel, and public attendees), 38-42 persons answered the questions that were provided. By far the least valuable information was identified as the medication list, but the most useful was any detailed information/opinion provided by the completing physician explaining the other information included on the form (e.g. cognitive testing, diagnosis). Though potentially more time consuming, highlighting the importance of this information to completing physicians may help them communicate more effectively to legal authorities and facilitate greater understanding of the physician’s opinion in guardianship cases.

